# Beauty Is Skin Deep; The Self-Perception of Adolescents and Young Women in Construction of Body Image within the Ankole Society

**DOI:** 10.3390/ijerph18157840

**Published:** 2021-07-23

**Authors:** Ruth Kaziga, Charles Muchunguzi, Dorcus Achen, Susan Kools

**Affiliations:** 1 Faculty of Interdisciplinary Studies, Mbarara University of Science and Technology, Mbarara 1410, Uganda; cmuchunguzi@must.ac.ug (C.M.); dorcus.achen@vub.be (D.A.); 2 RHEA, Centre of Expertise on Gender, Diversity and Intersectionality, Vrije Universitet Brussels, 1050 Brussels, Belgium; 3 School of Nursing, University of Virginia, P.O. Box 800826, 202 Jeanette Lancaster Way, Charlottesville, VA 22908, USA; smk9h@virginia.edu

**Keywords:** adolescent girls, body image, self-esteem, Ankole

## Abstract

Introduction: Adolescents and young women become increasingly aware of their bodies through images presented to them through social structures during their developmental stage. These images may drive them toward unhealthy behaviors including overeating, starving, and skin bleaching. This paper is part of a study that examined the Older Adolescent Banyankole Girl’s Response to the socio-cultural constructions of body image in The Ankole Region, Uganda. It aimed to understand the self-perceptions of adolescent girls of their body image within Ankole society. Methods: The study collected narrative interviews of 30 adolescent and young adult females (16–24) recruited from various institutions of learning as well as the Ankole community of southwestern Uganda. Results: Adolescent girls’ perceptions of beauty were influenced by pull and push factors that included beauty expectations, beauty comparisons, relationships, and dietary habits that keep them oscillating between traditional and contemporary beauty ideals. Findings suggest that young women could benefit from social shifting of focus from physical appearance to other valuable developmental assets. Conclusion: Government-sponsored programs that provide education and positive media messages may be beneficial to building the self-esteem of young women.

## 1. Introduction

Several studies have found significant links between well-being and positive body image in adolescent girls and young women. From a very young age, they are told that how you look is important to them and others who look at them [1,2,3]. Cultures all over the world put women and girls’ bodies at the center of intrigue based on connotations attached to beauty such as goodliness and sexuality. Studies have showed that young girls may obsess over their appearance making other aspects of development less important, such as education or independence [3]. According to [4,5], girls may adopt unhealthy eating habits such as skipping meals at school and at home so as to strive for the Western standard of beauty that values slenderness. The study reveals that when adolescent girls are still going through the bodily changes of puberty, this makes it even more challenging for them to achieve the societal standards of female beauty.

In adolescent development, there are certain aspects in society that are fixed on how young people behave rather than simply the changes of a growing body or cognitive structures. Individualistic cultures in Europe and South Africa place more emphasis on self-esteem [6]. In contrast, in collectivist societies such as Uganda, self-perceptions of body image can be based on the societal norms. Studies show that social expectations have an influence on young peoples’ ideologies [7]. The study shows that the existing beauty standards in Ankole play a role in how young girls feel about their bodies.

With embedded norms with relevance to society of how one should look, overwhelming pressure is put on girls to conform, which in turn affects their self-esteem when they do or do not meet these norms [8]. Adolescent and young women (16–24) who are at the age of self-discovery have formed their perceptions of beauty images that are usually unattainable based on societal influences [9]. Research shows that there is an association between the social environment and the behaviors, feelings, and thoughts of individuals. These thoughts are usually passed on from parents, peers, and overt messages that encourage the “appearance culture” [10].

### 1.1. Aims of the Study

This study focused on the first objective of a PhD study on older adolescent Banyankole Girls’ Response to sociocultural construction of body image in The Ankole Region, Uganda. Its purpose was to explore perceptions of body image and how adolescent girls responded to these perceptions in Ankole. A secondary aim was to explore the lives of adolescents and young women who are candidates for marriage and advanced society roles in Uganda, and live at the crossroads as to which society’s body image ideals they should ascribe to; either the traditional and conservative or the modern and liberal societies.

The study was guided by socio-cultural theory [11] supplemented by Foucault’s theory of the body [12,13] and Higgins’ self-discrepancy theory [14]. The socio-cultural theory elucidates the importance of societal norms and their influence on young people’s cognitive perceptions of the body. Adolescent girls and young women in the study are shown to react toward society’s view of appearance; for example, in the traditions put in place, we see the rural Ankole society encouraging female fattening. Furthermore, the media encourage women to adhere to expectations of beauty. Foucault’s theory of the body explains the ethos surrounding the female body image from the past notion of its sole biological purpose and sexuality, which has been used to oppress women [12,15]. Higgins self-discrepancy theory points out that young people’s self-perceptions are influenced by comparisons with others. 

### 1.2. Background and Significance

In societies across Africa, female beauty ideals have been used to explain perceptions of one’s fertility, gender role identity for women, and the distribution of economic and political power in society [16]. Perceptions regarding beauty and body types vary between cultures across the world and have changed significantly across history [6]. In the past, most African countries beauty standards of women reflected a husband and father’s wealth and power, and this standard is still sought after; for example, in the Efik of Calabar cross river state in Nigeria, fat women symbolize fertility and well-being [16]. While studies underscore that many traditional societies covet fat as a sign of wealth and health [16], others show that some of the contemporary societies in Europe, Asia, and North America encourage slenderness among young women as shown through media [3,9].

In pre-colonial times, Ankore was a part of the Chwezi empire the ruled the Great Lake’s region of East Africa, which is now in southwestern Uganda [17]. It was a traditional kingdom that was abolished in 1967 by President Milton Obote and has not been officially restored. While the kingdom was abolished, people continue to maintain this cultural identity [15,17]. This society has two groups subgroups within Ankole culture with a common ancestry: the Bairu and Bahima. Both ethnic groups’ standard of beauty is characterized by a slender nose, thin lips, finely-shaped heads, fat backsides, and fat legs [18]. According to [16,19], in Ankole culture, the beauty standard is fat. A fat body composition for the Ankole woman has been traditionally indicative of fertility, sexuality, and morality. Body image for Ankole women is also tied to moral norms [18]. Among the Bahima of Ankore, there is a beauty ritual of female fattening during adolescence to ensure that their women develop to be fat. Fattening among women engenders a great sense of respect within and outside of the family [16]. Fattening is similar to the past practice of the Bairu in Ankole, in which for preparation of marriage, young women are bulked up to the size of a millet basket [18].

While these traditional practices looked to fatness as a symbol of beauty in Ankole, the trend for young women has begun to shift in the contemporary world. Research and the press [4,20] have shown that over time, beauty trends in Uganda have changed due to Westernized views on what it means to be beautiful; therefore, this has encouraged many girls and young women to maintain an unhealthy body weight, as well as the practice of fattening. The young women have adopted the global standards that idealize being thin and curvy. Women and girls are starving themselves and bleaching their skin to match the Westernized images in the media [4]. These changing trends may put young women at risk for self-hatred toward their bodies [4,5].

Although young women of Uganda have more recently embraced the thin ideal of beauty from the West, tradition is still an important factor of growing up [4]. Some young women, especially those in the rural areas, still look to fatness as the beauty ideal. This has led to the failure of many rural girls to maintain a healthy body size, resulting in health risks such as obesity and cardiovascular disease [21]. Research suggests that girls who struggle with a negative self-esteem are more likely susceptible to harmful societal messages and struggle with body dissatisfaction [1]. A negative body image is not only connected to low self-esteem and decreased well-being, but it is also related to serious long-term psychological consequences, such as depression, suicidal thoughts, eating disorders, and poor performance in school [4].

## 2. Materials and Methods

A phenomenology research design was used, collecting and analyzing young women’s stories from their lived experiences [5,22]. By directly querying individuals about their lives, this research design allowed for the exploration of the range of subjective experiences young people have. Through triangulation of interview data with field notes of observations of the young women in their urban and rural community contexts, the researcher focused on past and current experiences of participants related to the influence of sociocultural factors and how they have influenced their thought process about their bodies over time [2].

The sample included 30 adolescent and young adult girls, both in school (high school and universities) and out of school in the age range of 16 to 24. In this study, 16–18-year-olds were considered to be in the developmental phase of late adolescence, while 19–24-year-olds were considered to be young or emerging adults. In Uganda, these ages include school-going people and those out of school and already married [23]. Young people both in and out of school were sampled, as it was assumed that those in school may be shown to be more influenced by peers and educators, while those with less or no form of formal education may be largely influenced by traditional norms and families [23]. Participants that identify as married are shown to be largely influenced by their spouse/significant other. This sample represented the nine districts of a southwestern region of Uganda. Among the participants, 5 adolescents and 7 young women were from the urban regions of Mbarara and Kampala, while 2 adolescents and young women were selected from each of the 9 rural districts of Ankole, respectively. All were of the Ankole culture.

Purposive sampling was used to recruit participants from universities, workplaces, and communities, where a notice was posted to encourage research volunteers to take part in the study. Snowball sampling was based on referrals from participants [24]. Participants from high schools were recruited by the head teacher; then, the selected students would refer other students. While recruitment from the community was based on telephone and face-to-face recruitment, those selected would then refer other participants. The inclusion criteria were that participants were between the ages of 16 and 24 years of age and had to belong to the Banyankole tribe.

Data collection procedures. Research assistants and the first author conducted semi-structured interviews in communities and workplaces of participants in the location they preferred. Interviews lasted between forty-five minutes and an hour and half. The interview questions were organized around the objectives of the study and included questions such as “How would you describe a beautiful woman in the Ankole culture?” “Considering your body and the way it looks, how do you fit within the Ankole culture beauty standards?” (see Appendix A for Interview Guide). Saturation was reached when participants gave no new information and themes were exhausted. Field notes were made on observations during the interview by research assistants and the first author, including the context where the interview took place and the nonverbal behavior of the participants in response to the questions. Observations were made on how participants approached the interview sessions and their reactions toward certain interview questions, especially those that triggered them. Observations were also made at the Ankole museum of Uganda, where different artifacts on traditional beauty in Ankole are displayed. The data were audio recorded and transcribed verbatim with participants identified with numbers to protect confidentiality.

Data analysis. Transcripts and field notes were coded manually to formulate themes using the process of thematic analysis as described by [25]. The goal of thematic analysis was to derive themes that were able to explain and address the study aims [26]. By using the six phases of thematic analysis by Braun and Clarke (2012), interview transcripts were read multiple times to develop an understanding of the participants’ experiences, highlighting information that stood out and making notes against the data. 

Coding was done manually by the first author on a hard copy of the transcript. A hybrid approach to analysis was used including inductive coding directly from the data and deductive coding from a priori concepts developed from the literature [1,2], including relationship influences and beauty comparisons. Similar codes were color coded, categorized, and eventually merged into themes. For example, beauty descriptions of the young women were clustered into the beauty expectations theme. In this way, patterns were identified using codes and categories, and more abstract themes were developed based on inductive codes, existing literature, and theoretical underpinnings [25]. Then, they were reviewed again to assure that they were relevant to the study aims, and where appropriate, some subthemes were merged into larger themes. Lastly, the themes were labeled in a concise, clear manner.

## 3. Results

**Figure 1:**
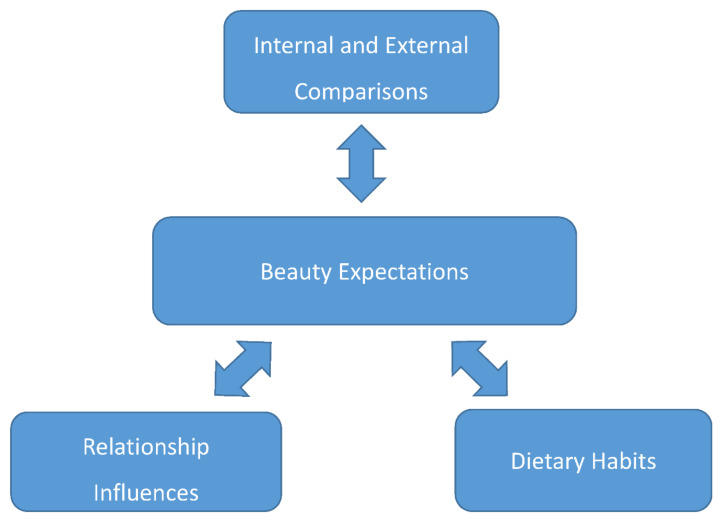
Relationship between beauty expectations and subthemes.

The most salient, central theme that emerged related to the perceptions of Ankole adolescents and young women on body image was beauty expectations. It was by far the most prominent theme in participant responses. Relevant subthemes were discreet, yet interrelated and organized around the central theme of beauty expectations, including internal and external comparisons, relationship influences, and dietary habits (see Figure 1). The central theme and subthemes will be presented with data illustrations.

### 3.1. Beauty Expectations

Findings revealed that participants have preconceived perceptions of beauty, and these perceptions are shown to change over time because of the amount of exposure which is seen through societal influences that include media, peers, parents, and culture. Participants’ views and expectations are shown to change when they left rural areas for urban areas to work or study, while participants who remained in rural settings are shown to have been largely influenced by traditional Ankole culture. Both Bairu and Bahima participants described a beautiful woman as fat with wide hips and big buttocks and decently dressed. All participants from rural Ankole described a beautiful woman from Ankole as fat with a small waist and big buttocks. She should be dark-skinned, have short black hair, long arms with slender fingers, big legs, but also at the same time, a beautiful woman had to be decently dressed.

When asked to describe a beautiful woman in Ankole, participant 3 explains that:

*She is fat! The Bahima love fat! That is why, the girl is given milk for her to become fat, get stretch marks, in the past, girls used to cut off their hair and dress up with beads to bring the allure of beauty and pride. (Participant 3 is a 19-year-old from rural Ankole)*

However, 10 of the participants identified that beauty and ethics coincide together; one cannot be beautiful and yet behave badly; morals play an important role in beauty expectations. One young woman said:

*Beauty is not only skin deep, but it is also aligned with proper manners and etiquette, big and beautiful women, take their time, are graceful in their walk and talk, and are not in a rush because they know what they are doing. (Participant 1 is a 24-year-old from rural Ankole)*

While the two subgroups in Ankole had similar beauty expectations of a young woman, there are different traits. Participants who identified as Bairu described a beautiful woman in Ankole as one who is energetic with tough features that indicated that she worked well as a farmer. Participants who identified as Bahima explained that culturally, a beautiful woman is one with a soft and fat body, indicating that there should be no fieldwork.

Participant 8, a 20-year-old Mwiru from the Mwiru subgroup from Mitooma, a rural area in Ankole, explains that culturally, a beautiful girl is one that:

*Among the Bairu, a beautiful girl that you can see must be tall. Maybe she is black or she is brown. She is energetic. By energy I mean, we usually engage in farming, so we know how to dig so we have tough hands.*

Participant 3 is from Kiruhura, a rural area largely populated by the Bahima; she describes a beautiful woman as one:

*A beautiful woman usually has a soft and fat body, with small baby-like fingers, usually, they will not engage in field work, they have delicate-like features, it shows that she’s well taken care of by her family.*

Observations made at the Ankole museum in Mbarara supplemented participants’ perceptions of beauty expectations of a young woman in Ankole. These were shown in a historical portrait of a young woman in Ankole. These were physical traits derived from nature and the environment around. For example:

*A beautiful woman in Ankole has eyes like stars, snow-white teeth, smooth and well-built arms, reed-like fingers, hair-like tree canopy, a neck of a water jar, breasts like a young fruit, a waistline of a wasp, hips of a churning gourd, legs like a banana stem and finally a baby’s foot. (A portrait of a beautiful woman in Ankole at the Mbarara Museum)*

When asked if they met these beauty expectations, participants who did not fit these expectations considered themselves as not beautiful. Results show that 20 out of the 30 participants reported a lack of confidence toward their bodies brought on by the beauty expectations in Ankole. This was based on a couple of interview questions that queried their views on whether they met these standards (see Appendix A for interview questions). Participants from urban centers and contemporary Ankole reported having self-hate and low confidence because while in rural Ankole, a fat woman was beautiful, this was not the case in the cities. A young woman reported all her friends walk around with curvy bodies (in this case a small waist and large buttocks and hips). Her perception is that she is ugly because she cannot gain weight and have a body similar to those of her friends.

Interviewer: *Do you think you fit in these beauty expectations in Ankole?*

Participant 5 is a 21-year-old from rural Ankole:

*Unfortunately, I don’t. I am a very slender girl with tiny hips and a very small bum. I have a boyish body that I hate so much. My friends and family have told me to drink a lot of milk and eat more food but until now I have not gained weight.*

Most participants from towns and cities reported that beauty expectations of a young woman in Ankole meant that she had to be medium in size, have a slender nose, chocolate skinned, and long curly hair, while four of the participants described a beautiful woman as one with light skin with a slender body. All the 12 participants from the cities describe a fat woman as one that was unhealthy and lazy. Out of the 12 participants who lived in the city, seven had been born and raised in the city, while five had left the rural areas to find better jobs and education in the city.

Participant 7, a 20-year-old woman from urban Ankole, voiced her desire to be slender; she believes slenderness is synonymous to healthy. She said:

*You see our parents think a very fat woman is a beautiful woman in Ankole. My parents insist that I look beautiful when I have gained weight; however, when I go back to the city my friends will make fun of me and call me a big mama so I have a hard time keeping a healthy weight. If I am not starving myself, I am overeating.*

When asked about the perfect body in Ankole, the majority of participants identified a medium body as the perfect body. Participants who reported to not fit the ideal body were more likely between the ages of 20 and 22, and they described having no self-assurance toward their bodies, reporting dangerous eating habits such as using diet pills and/or overeating.

When asked if they believed they fit in the perfect Ankole ideal, participant 10, a 21-year-old from an urban area, believed that:

*I don’t fit that perfect ideal body, I have always had a protruding tummy, and yet beautiful girls should have wasp waists and tiny tummies, I have tried starving myself, but a friend of my mine [said] to always drink lemon water, so I hope I see changes very soon.*

However, results show that there were contradictory responses from young women and girls based on where they lived. Participants who described themselves as having the “right body” were more likely still living with their parents and had not been influenced by outside factors, such as the media. These participants perceived themselves as fat and dark-skinned, which is an ideal body sought after in rural Ankole despite their age. However, one should note that participants from urban centers who perceived their bodies as “just right” identified not too slender and not too fat but “medium” and chocolate skinned as the perfect body. This seemed to be based on influences such as the media and bodies of that of Beyoncé and Anita Fabiola (celebrities) and the fashion industry. Participant 12, a 20-year-old who lives and studies in the capital city, describes it as a place highly Westernized. She believed that everyone was aspiring toward what they see on TV and social media. She says:

*I still believe that light skin is the beautiful and skinny just average weight but not very skinny, like a medium-size, small waist, and a relative bum not like mine (laughing) don’t go crazy. In terms of hair, I don’t have any preference because people look nice with short hair, long hair but I still believe in the whole light skin and the curvy body as beautiful.*

In Uganda, people’s bodies are commonly described using figures to depict body figures. These figures ranged from one to nine. This is a continued and common practice for young and older people when describing one’s body. The figure one describes a slender body without curves that is usually masculine, while the figures six depicts a pear-shaped figure, while eight describes an hourglass figure, figure and figure nine refers to when one has a larger upper body than the lower body. So, when describing what is considered to be beautiful, participants had various yet similar descriptions of a beautiful woman in Ankole. In this case, when participants described their appearance, they associated their bodies with figures one to nine. Figures six and eight described the sought-after body while figures one and nine were used to describe an “ugly body”. Participants who did not identify with figures “six” and “eight” reported negative feelings toward their bodies. A “figure six” body was described by participants to mean that one has a slimmer upper body and a larger lower body with “big hips and buttocks”, while “figure eight” meant one’s body had bigger breasts a “wasp”-like waist and a large behind. Figures one and nine were described by participants as the least desirable bodies.

*“Figures one and nine” are the ugliest, where one usually doesn’t have curves, hips, and a bum they’re sticks and built like boys, no man would want a woman who is built like a man or boy because then who will be the man in the relationship.*

*“Figures six and eight” are very feminine, my friends and mother tell me that when you have big hips, giving birth will be very easy so having big hips is very important, this is why I drink a lot of milk to ensure that my hips continue growing bigger.*

Again, one should note that the results show contradictory statements from participants from rural and contemporary urban Ankole. Participants from rural Ankole identified a fat woman as the ideal body type based on images from their parents and cultural traditions, while participants from contemporary Ankole identified a slender and curvy woman as the ideal body type based on images in the media.

*I think* Nicki Minaj *has a great body, she may be cosmetically enhanced but I think that’s what a perfect body looks like. (Participant 12)*

### 3.2. Internal and External Comparisons

Results indicated that participants made comparisons of their bodies with what they see in the media, among friends and peers, in their homes and their community. Comparisons include the young woman’s own thoughts about how their body compares with others (internal beauty comparison), as well as those stemming from criticisms or comments from those in their social network (external beauty comparisons). These comparisons have been shown to influence their self-perceptions of beauty, in turn encouraging feelings of self-hate and lack of confidence.

When asked when body criticism began in their lives, most stated that they began to notice differences with their bodies and other girls and women when they turned 12 and 13 years old. This was usually pivotal when they began high school or started their first period. Results showed that their comparisons with friends and peers was notable to them in evaluation of their own beauty. Participants reminisced on their first time they developed a negative body image. However, it should be noted that body preferences differed with age. For example, for participants whose bodies began to change rapidly during puberty with enlarged hips and breasts, they developed a negative body image and were more likely engaged in risky relationships because such features meant that they had become women and were mature enough to engage in such behavior such as sex and drinking alcohol.

When asked when body criticism from peers and others in the community began, participant 12, born and raised in the city, reflected back when she was just 13 and how she felt about her body:

*When I turned 13, my friends would point out that my hips were of a woman and that my breasts were big. Whenever I would walk back home, taxi men would tell me that my body was that of a grown woman and that I should just stay home and get married and have children. I hated walking back to school fearing that they would touch me and make more stupid comments.*

Participants over the age of 16 in significant relationships who described their bodies as too thin and reported having negative feelings and self-hate toward their bodies resorting to overeating, because a small body meant that one was not feminine enough, and they were associated with children. Small breasts, small hips, and a small behind meant participants were not yet young women. One 21-year-old woman from the city described her time growing up, when all her friends started showing off their growing breasts, yet she had not grown an inch. She felt insecure when comparing herself with her friends:

*When I turned 16, my breasts did not grow like the rest of my friends, I tried everything they told me for them to get bigger like I rubbed fruit on my breast every night hoping that they grow, my friends would make fun of me until they eventually came when I turned 18.*

Similarly, the study showed that the perceptions of peers and others of their bodies affected the way the participants felt about their body image. Most participants reported that friends, intimate partners, and coworkers had their perceptions of a beautiful woman, and these perceptions oscillated from a fat dark-skinned woman to a slender light-skinned woman to a curvy, medium weight and chocolate-skinned woman. One young mother reported that her body has never looked the same since she gave birth. She hates that her waist has gotten bigger and when asked how her partner feels about her, she says that:

*When I got married at 20, I had a very small waist and my husband made it a point reminding me that it’s one of the reasons he fell in love with me. But now I have added a few kilograms especially around my waist after having two children, I try everything from slimming pills to drinking lemon tea every day to get my waist back. It’s honestly frustrating and I hate going to parties with my friends because I don’t feel beautiful anymore.*

All participants in the study endorsed comparing themselves to others, thus shaping their perceptions of a beautiful body in Ankole.

### 3.3. Relationship Influences

Contrary to the majority of participants being greatly influenced by their own perceptions of what a beautiful body should be, those who had positive relationships with parents and peers and relied largely on their religious beliefs did not perceive any body type as ideal but believed that a beautiful woman was one that was kind, decent, and God-fearing. These participants despite their age brackets described beauty as authentic. A few of the participants who identified as religious reported high self-assurance compared to those who did not consider themselves religious. One young woman considered herself as very religious, believing that the church can act as a solace for hope and love. She believes that her love for herself and her body regardless of how she looks is brought on by her faith in God. When asked how she feels about her body she says:

*I am confident in my skin, God made us in his image, so everyone is beautiful; besides, we all can’t look the same. I always pray to God whenever I have feelings of self-doubt and hate.*

Participants who listed positive relationships with their family, peers, and friends described their perceptions of beauty to be genuine and more authentic. Many participants reported having more positive relationships now that they are older. They also reported self-assurance and a positive outlook toward their bodies brought on by these relationships.

### 3.4. Dietary Habits

All participants reported adopting dietary habits based on the perceptions they have of their ideal body. However, it should be noted that dietary habits were consistent with expectations in the two contrasting societies in Ankole. Dietary habits of participants from rural Ankole encouraged weight gain and practices such as the all-dairy and carbohydrate diets. For example:

*The Bahima girls only drink milk; fresh warm milk to be specific; they mix the yogurt with millet flour porridge, the milk will fatten the girl because the nourishment is of both carbohydrate and protein.*

Dietary habits of participants from urban centers and towns in Ankole encouraged weight loss or overeating. Diets consisted of refined food, evasive diets such as juicing, plant-based diets, and no-carb diets. Participants who adopted such dietary habits were most likely between the ages of 20 and 24 years and were either working or at the university. The majority of the participants who adopted these diets reported low confidence and self-hate toward their bodies brought on by peer pressure from friends and colleagues. An example:

*During the lunch break at my workplace, my boss has a habit of pointing out our imperfections, like one time she told a friend of mine that she needs to eat more vegetables because she has a big tummy, so currently she’s on a no-carb diet.*

A few participants at workplaces and school described practices of skipping meals and taking part in complicated diets such as all-green diets and no-carb diets as they struggled to attain the desired body. This often led to anxiety, stress, and underperformance at school and work.

## 4. Discussion

This paper aimed to investigate adolescents and young women’s perceptions of a beautiful woman in Ankole and how these perceptions have appeared to affect their feelings toward their bodies. Vygotsky’s Sociocultural Theory of Cognitive Development (1934) and feminist theory on body image development [27] address the existing socio-cultural influences on the female body in Ankole brought on by ever-changing globalization. This is manifested in the contradictory perceptions of beauty expressed in the voices of these young women in Ankole. There are clearly both psychological and physical risks and benefits of different types of influences on beauty expectations. The long-term goal of the study is to develop an intervention to promote body positivity and high self-esteem in young women in Uganda. In order to realize this goal, the results of the study will significantly inform the conceptualization, design, and implementation of future interventions.

The socio-cultural theory posits that existing social norms such as those related to peer perceptions, parents, and the media influence behavior and thought processes among young people through the messages that are conveyed [11]. Adolescents aspire to fit in, and this may encourage their need to seek approval through adopting behavior that is supported by social agents [10,11].

The socio-cultural theory largely explains how young people learn from the interactions they have with important social agents in their lives, but it fails to elucidate the feelings young people develop when they make comparisons with existing beauty ideals in society. This study adopted the self-discrepancy conceptualization by Higgins (1987) that explains that when individuals, in this case adolescents, make comparisons with a person, most likely using images presented in society and find an incongruity, consequences arise. According to the literature [3,28], adolescent girls will modify their eating behaviors to fit the ideal standard of beauty presented in that society [3]. College students reported having developed eating disorders brought on by the slender ideal presented in the media through magazines and social media [28]. The self-discrepancy theory highlights the feelings young people develop when they make comparisons with unattainable images presented and may lead to the dissatisfaction and satisfaction they have with their body images. The study adopted the feminist theory of the body that looks to existing structures in society that have long focused on the body image of women with cultures all over the world presenting the desired image [12,26]. These cultures have traditional practices in place that put women’s bodies at the center of intrigue. These practices include female genital mutilation in many African states, feet binding in China, and female fattening in the northern and east of Africa.

Adolescent girls and young women’s perceptions of beauty may differ depending on what type of society they live in [29]. Beautiful women in Ankole as described by the majority of young adult girls are fat—this is shown in their description of a beautiful woman, where they compare her body to objects in nature. This finding is supported by literature [16,21] that claims the African cultural preference of a beautiful woman is fat. Studies by [21] show that this perception has encouraged young women to adopt unhealthy eating habits such as overeating and carb-loaded diets to attain this ideal. This has led to an increase in obesity and cardiovascular diseases among young women and girls.

However, it should also be noted that there has been a shift in paradigm where fatness was and is in some parts of Ankole synonymous with beauty. Findings show that while the perception of a beautiful woman in Ankole is fatness, this is not experienced by some in urban places in Ankole society that are more Westernized. Some of the young adult Ankole girls from urban areas described a beautiful woman as one that was medium with average-size hips and a large behind, which can be described as curvy with Eurocentric features of beauty. This was common in older and highly educated participants who reported a low level of self-assurance and confidence toward their bodies based on their descriptions. According to studies [1,7,20,29], there is a shift from the traditional perceptions of feminine beauty to more contemporary aspects of feminine beauty.

While the literature on some African cultures of the female body such as those of west Africa and south Africa [16,20,30] show that fatness is synonymous to beauty, this study contradicts such literature showing that some of the young adult girls from urban areas of Ankole are driven by the effects of globalization and have beauty expectations that are similar to Westernized views, which are largely Eurocentric. Many young women and girls from urban areas of Ankole have adopted a Westernized view of beauty that is the thinner and curvier body. Most of the comments made by others focus the participant on “shedding” the extra weight by adopting unhealthy eating habits such as skipping meals or adopting a no-protein diet to meet the unrealistic expectations of the body ideal.

In this study, rural districts of Ankole show that adolescent and young adult girls of the ages of 16–24 make internal and external beauty comparisons. Young women in rural Ankole may adopt the dietary habits of the Ankole society to strive for the fat beauty notion. Results showed that because slenderness is associated with being weak and unhealthy, young women who made positive comparisons to others with larger bodies adopted dietary habits that encouraged weight gain. The studies [16,19] look to the Ankole culture and the emphasis on a dairy diet of milk that acts as a source for enlarging their physical features. This supports the claim in [21] that women in Uganda seek extra weight to appear more desirable and similar to the rest of women in their culture, encouraging poor eating habits that may place them at risk for health problems such as obesity and cardiovascular diseases.

The findings also reflect how relationships in society influence beauty perceptions of young women and girls. According to [27,29], body dissatisfaction is greater for women in socially valued roles such as employment and romantic relationships. The finding resonates with a study on peer and parental relationships where the author found that parent and peer comments on appearance encouraged unhealthy body image [2]. Young people may dwell on such comments and in turn may flounder in their work [15]. These findings verify those of [2], indicating that young women and may internalize unrealistic beauty perceptions from significant others and employers, placing young adult girls at risk of developing unrealistic perceptions of their bodies based on the poor judgements toward their bodies brought on by those close to them.

Similar to [2,9,31,32], this study suggests that socio-cultural influences affect the way perceptions of body image and beauty develop. While most socio-cultural influences such as parents, peers, religion, and the media reported in the study tend to create a negative body image based on unrealistic perceptions of beauty, the young women in the study reported that these socio-cultural influences also encouraged their positive perceptions of their bodies. The authors of [33] explain that when surrounded by people who constantly focus on what is inside one’s mind and not the outside, a person’s perception of beauty tends to focus more on the mind. This finding suggests that positive relationships with affirmations may encourage positive perceptions of the body.

### Limitations

The sample was limited to 30 participants in particular districts in Ankole to provide their experiences with the existing social expectations of the female body. Therefore, the findings are limited in their representativeness of young women in these areas. The participants included were purposively selected; thus, they are not representative of the larger population of all adolescent girls and young women in Ankole. Girls of 18 and below tended to be less expressive than young women older than 18, so many of the narrative illustrations were for participants above 18 years old. Therefore, the findings may not represent all experiences of adolescent girls and young women. Future research on a larger population across the different regions within Uganda is encouraged, given that Uganda is a multi-culture state with differing beauty ideals.

## 5. Conclusions

This study has shown that adolescent girls and young women’s perceptions of beauty largely stem from socio-cultural influences; the study shows that young people will seek to attain unrealistic body shapes and sizes largely because the societies in which they live have created these images. Perceptions of these unrealistic images are shown to be shaped by socialization to outside influences such as the media. It can be argued that perceptions born out of such influences encourage unrealistic goals of body appearance, in turn affecting young people’s self-esteem. Findings suggest the need for a reframing of the emphasis on other positive developmental assets of young women rather than on their physical appearance. Government campaigns with supportive curricula and media messages that focus on building the self-esteem of young people could positively contribute to fostering generations of young women who are full of self-confidence and national pride.

## Data Availability

Not applicable.

## Appendix A

Interview questions (Narrative interviews) for young women and girls.

1. How would you describe a beautiful woman in the Ankole culture? (Introduce the topic)
2. In your own opinion with body image, how do you fit within the Ankole culture beauty standards? (Probes for positives and negatives)
3. As a young person growing up in in this society, have you and other young women of Ankole compared yourselves to young women and girls in media (social media that is Facebook, Instagram, snapchat, and the local media) and the media?
4. In your own experience, how do young women and girls talk about their bodies when in each other’s company? (Probe: How often?)
5. As young woman growing up, have you had positive/negative attitudes about dark skin and light skin?
6. Why do you think women and girls are much more likely to engage in body appearance than men and boys? (Probe: Why women and girls and not men and boys? Why men and boys and not women and girls?)
7. As a young man or boy, how would you describe a typical female body you desire and dislike? (Probe for appreciation and shaming conversations over female body image: What reactions do you have to those kinds of conversations? Do you ever have conversations like this?)
8. Have you ever been in a relationship? (Probe for (1) positive appreciation of her body by the fiancée. (2) Positive appreciation for self-perception of her body due to the comment made by the fiancée/partner/fiancée. (3) Shaming comments of her body made by her fiancée/partner/boyfriend. (4) Her self-perception of her body from the shaming comments made by her fiancée/partner/boyfriend.)

Facilitators and Barriers to Body Image Among Adolescents Girls and Young Women

1. When did “body criticism” begin in your life?
  - What was happening with your body during this time?
  - What messages did you receive about your body and appearance?
  - What did people say about your body?
  - What kinds of non-verbal messages did you receive?
2. What do you see when looking at yourself today?
  - When do you focus on the physical aspects that you think need to be changed?
  - When do you feel comfortable with what you see? What traits do you like?
  - How do others respond to your appearance now? Does this have an effect on your feelings of body criticism or acceptance?
  - What circumstances, emotions, thoughts, or other factors might impact whether you feel positively or poorly about your body in your current daily life?
3. What kind of impact does “body criticism” have in your life today?
  - To what extent does “body criticism” limit, constrain, hurt, or otherwise feel oppressive to you?
  - Does it cause harmful body practices such as over or under-eating, over or under-exercising, etc.?
  - How much time do you spend thinking about your body and appearance in interactions with others? How do you think body criticism affects your interactions with others?
4. How often do you engage in conversations about body image, and how do you think they affect you?
5. As a young person growing, how did your attitude about skin tone develop throughout your life, particularly your childhood?
6. Is there a body shaming issue at school, in your homes, from the community and intimate relationships? (Probe: If you feel there is body shaming, what should be done about it?)
7. In your own experience, because of the media, is there a specific definition of a ‘perfect body’ that young women and girls want to achieve? (Probe: What struggles do you go through to achieve this body?)
8. In your own experience, does the Ankole traditional society create an unattainable body image for young women, and how do you feel about the way the media portrays women and men?
9. In your own experience, what do we do about the ways we view our bodies a norm that has been ingrained into our psyche for many years?
